# Induction of Peroxisomal β‐Oxidation as a Critical Mechanism for Ethanol‐Induced Hepatic Triglyceride Accumulation

**DOI:** 10.1096/fba.2024-00211

**Published:** 2025-05-02

**Authors:** Yida Zhang, Wei Zhang, Yicong Li, Haoya Yao, Yaoqing Wang, Xiao Zhang, Jia Zeng

**Affiliations:** ^1^ School of Life Science Hunan University of Science and Technology Xiangtan Hunan P. R. China

**Keywords:** acetaldehyde, alcohol, catalase, fatty liver, peroxisomal β‐oxidation

## Abstract

Excessive oxidation of ethanol has been well known to induce hepatic triglyceride accumulation, while the underlying pathogenic mechanisms are not fully demonstrated. The peroxisomal catalase–hydrogen peroxide complex system plays a role in the metabolism of ethanol, while the potential origin of hydrogen peroxide involved in ethanol oxidation by this system is not determined. As peroxisomal fatty acid β‐oxidation generates hydrogen peroxide and can be induced under ketogenic conditions, we hypothesize that induction of peroxisomal β‐oxidation might accelerate ethanol oxidation through increasing the supply of hydrogen peroxide. The study reveals a novel mechanism by which upregulation of peroxisomal β‐oxidation stimulates ethanol metabolism and induces liver triglyceride deposition in animals. Excessive oxidation of fatty acids by peroxisomes generates considerable hydrogen peroxide in mouse liver, which significantly enhances liver ethanol oxidation and induces hepatic triglyceride accumulation through elevating mitochondrial NADH/NAD^+^ ratio and suppressing mitochondrial fatty acid oxidation. Specific inhibition of peroxisomal β‐oxidation suppresses ethanol oxidation in the liver and attenuates ethanol‐induced hepatic steatosis in fasting mice. It is proposed that induction of peroxisomal β‐oxidation serves as a critical mechanism for alcohol‐induced hepatic lipid accumulation in animals under ketogenic state, and targeting peroxisomal β‐oxidation might be a potential pathway in treating alcoholic fatty liver through reducing the supply of hydrogen peroxide and suppressing peroxisomal ethanol oxidation.

## Graphical abstract

Prolonged fasting and fibrate treatment induce peroxisomal β‐oxidation and lead to excessive generation of hydrogen peroxide, which stimulates ethanol metabolism and significantly increases the formation of acetaldehyde; further oxidation of acetaldehyde into acetate remarkably elevates the mitochondrial ratio of NADH/NAD^+^ and causes feedback suppression of mitochondrial FAO, which ultimately leads to the accumulation of TG in the liver.

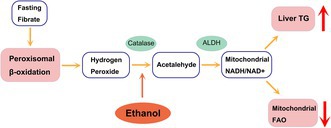

**Abbreviations**
βOHB: β‐hydroxybutyrate
AcAc: acetoacetate
ACOX‐1: acyl‐CoA oxidase‐1
ADH: alcohol dehydrogenase
ALDH: acetaldehyde dehydrogenase
AMPK: AMP‐activated protein kinase
ATGL: adipose triglyceride lipase
CFB: clofibrate
ER: endoplasmic reticulum
FAO: fatty acid oxidation
FFA: free fatty acid
LC‐CoA: long‐chain acyl‐CoA
MEOS: microsomal ethanol oxidation system
mTORC1: mechanistic target of rapamycin complex 1
PEX2: peroxin 2
PPARα: peroxisome proliferator activator receptor α isoform
SREBP‐1c: Sterol regulatory element‐binding protein 1 couples
TCA cycle: tricarboxylic acid cycle
TDYA: 10,12‐tricosadiynoic acid
TG: triacylglyceride

## 1 Introduction

Excessive ingestion of alcohol has been well known to induce hepatic lipid accumulation, which plays a critical role in the development of steatohepatitis, fibrosis, and liver cancer [1, 2]. The metabolism of ethanol is complex and catalyzed by multiple oxidation systems; it is generally accepted that the major pathway for the oxidation of ethanol to acetaldehyde in mammalian liver is catalyzed by alcohol dehydrogenase (ADH), which is based on the fact that ethanol elimination is abolished nearly completely by the administration of inhibitors of ADH [3, 4, 5]. It is proposed that the increase in hepatic TG caused by ethanol is attributed to the oxidation of ethanol by ADH and the elevation in liver NADH/NAD^+^ ratio, which causes suppression of mitochondrial fatty acid oxidation (FAO) and esterification of fatty acids [6, 7]. However, the ADH mechanism later is challenged based on the following facts. First, ethanol induces hepatic TG accumulation only under the condition of a fasted state [8, 9], and the ADH metabolic pathway does not play a primary role in ethanol oxidation under fasted conditions [10]. Second, the administration of pyrazole, a specific inhibitor of ADH, does not abolish ethanol‐induced hepatic TG accumulation in fasted rats [11]. Therefore, the ADH pathway in ethanol metabolism might not be crucial in inducing liver lipid deposition; there might exist an alternative mechanism that leads to diminished fatty acid oxidation and TG accumulation in ethanol‐treated animals.

It is notable that ethanol can be efficiently metabolized in peroxisomes because this molecule is a peroxidic substrate for catalase and can be well oxidized by the catalase–hydrogen peroxide complex system [12, 13]. As catalase is abundant in liver peroxisomes, the supply of hydrogen peroxide will be a critical factor in mediating the turnover of ethanol [14, 15]. It is rational to assume that catalase oxidation of ethanol might be prominent when the supply of hydrogen peroxide is sufficient.

To explore the potential source of hydrogen peroxide that might be involved in ethanol metabolism in peroxisomes, we shed light on peroxisomal β‐oxidation system, a FAO system that located in mammalian peroxisomes and acted on long‐chain and very long‐chain fatty acids [16, 17]. Although the crosstalk between peroxisomal β‐oxidation and ethanol induced hepatic lipid accumulation in animals is not established so far. Evidences suggest that the metabolism of ethanol by catalase system can be stimulated by free fatty acids (FFA), and most possibly due to the increased hydrogen peroxide generation from peroxisomal fatty acid β‐oxidation [18, 19]. As liver peroxisomal fatty acids β‐oxidation is induced in animals under the conditions of fasting, diabetes and high fat diet feeding [20, 21, 22], we hypothesize that peroxisomal β‐oxidation might play a dominant role in ethanol metabolism and also be involved in ethanol induced hepatic lipid accumulation under ketogenic conditions.

This study investigated the potential role of peroxisomal fatty acid β‐oxidation in ethanol metabolism and explored the potential mechanism by which induction of peroxisomal β‐oxidation caused hepatic lipid accumulation in ethanol treated mice.

## 2 Experimental Procedures

### 2.1 Materials

Coenzyme A sodium salt, acetyl‐CoA, Percoll, and defatted bovine serum albumin (BSA) were purchased from Sigma (St. Louis, MO, USA). Clofibrate (CFB) and 10,12‐tricosadiynoic acid (TDYA) were from Tokyo Chemical Industry (Tokyo, Japan). All other chemical reagents used were of analytical grade.

### 2.2 Animal Studies

C57BL/6J mice at the age of 8–10 weeks were from Slac Laboratory Animal Co. Ltd. (Changsha, China). Standard rodent diet (12% fat by calories) was supplied by Slac Laboratory Animal Co. Ltd. (Changsha, China). All the animals were housed in single cage with free access to food and water under controlled temperature (22°C) and light. To investigate whether induction of peroxisomal β‐oxidation might stimulate ethanol oxidation, CFB at a dose of 200 mg/kg/d was administered to the mice by gavage, once per day at 5 pm for consecutive 7 days. On Day 8, after a period of 6 h without food, both the ethanol control group (Ethanol) and CFB group (CFB + Ethanol) received ethanol at a dose of 3 g/kg, given as a 30% solution (by volume) in water by gavage. For TDYA intervention, TDYA was administered to the CFB‐treated mice by gavage at 100 mg/kg to suppress peroxisomal ACOX‐1 (CFB+TDYA+Ethanol). Controls group (N) received equal volumes of water. To study the effect of TDYA, a specific inhibitor of ACOX‐1 on ethanol induced hepatic TG deposition in the fasted mice, C57BL/6J mice was fasted for 24 h, after that the TDYA group was treated with TDYA at 100 mg/kg by gavage and the control group was administered with equal volumes of water, 1 h after pretreatments, both the control group (Ethanol) and TDYA group (TDYA‐Ethanol) were treated with ethanol at a dose of 3 g/kg, given as a 30% solution (by volume) in water by gavage. Normal group (F) received equal volumes of water. Six hours after the experiments all the mice were bled from the eyes and then sacrificed under anesthesia. Livers were removed quickly and stored in liquid nitrogen immediately. Liver histological analysis was performed according to the protocol as described previously [23]. All the animal studies were approved by the Animal Care Committee of Hunan University of Science and Technology.

### 2.3 Isolation of Mitochondria and Peroxisomes

Mitochondria were isolated by differential centrifugation of liver homogenate [24], peroxisomes from mouse liver were isolated by differential centrifugation and further purified by a Percoll gradient according to the methods as described previously [25].

### 2.4 Quantitative Real Time PCR

Real Time PCR was performed according to the procedures as described previously [26]. The used primers were: ACSL1, 5′‐TCCAAAAGGAAAGAGGCGGA ‐3′ (F) and 5′‐TCCTCAGAAACGTCAGCACT‐3′ (R); ABCD1, 5′TGAAGGAAGAGGAGCTGGT ‐3′ (F) and 5′‐TGGAACATCTCGTACACCCT ‐3′ (R); L‐BP, 5′‐AAATACAGAGATACCAGAAGCCG‐3′ (F) and 5′‐AAGAATCCCCAGTGTGACTTC ‐3′ (R); Thiolase, 5′‐CCTGACATCATGGGCATCG‐3′(F) and 5′‐AGTCAGCCCTGCTTTCTGCA‐3′(R); ACOX‐1, 5′‐CGTTACGAGGTGGCTGTTAA‐3′ (F) and 5′‐GCATCCATTTCTCCTGCTGA ‐3′(R). RNA expression levels normalized to 18S rRNA were expressed using the comparative delta CT method.

### 2.5 Biochemical Analysis

Plasma free fatty acid concentration was determined using a kit based on ACS‐ACOX method (Wako, Osaka, Japan). Plasma TG was determined by commercial kit according to the manufacturer's instructions (Wako, Osaka, Japan). Blood glucose was determined by a glucometer (Lifescan, Johnson and Johnson). Plasma insulin was measured by a mouse insulin ELISA kit from Merck Millipore (Billerica, MA, USA). Plasma and liver content of ethanol and acetaldehyde were determined using assay kits from Sigma. The content of acetate was determined enzymatically according to the method of Knowles [27]. Plasma and liver β‐hydroxybutyrate (βOHB) and acetoacetate (AcAc) were determined enzymatically according to the method as described previously [28], total ketone body (KB) was expressed as the sum of βOHB and AcAc. Liver NADH and NAD^+^ contents were determined by an assay kit from Sigma. Liver LC‐CoAs was extracted and determined by the method of Tubbs and Garland [29]. Liver TG was extracted by the method of Bligh and Dyer [30] and determined with a commercial kit (Wako, Osaka, Japan). Liver alcohol dehydrogenase (ADH) was assayed according to the method as described previously [31]. Liver aldehyde dehydrogenase (ALDH) was assayed according to the method developed by Blair [32]. The activity acyl‐CoA oxidase was measured according the method of Zeng [23]. Peroxisomal β‐oxidation was assayed by acyl‐CoA‐dependent NAD^+^ reduction in the presence of KCN [33]. Liver hydrogen peroxide and catalase activity were determined by commercial kits (Sigma, St. Louis, MO, USA). Protein concentration was measured by Bio‐Rad DC protein assay kit (Hercules, CA, USA).

### 2.6 Statistical Analysis

Data are presented as mean ± SD. *n* = 8 for all the groups. The significance of differences was evaluated using one‐way ANOVA with Dunnett's T3 test or Student's test by SPSS 18.0. *p* < 0.05 was considered statistically significant.

## 3 Results

### 3.1 Induction of Peroxisomal β‐Oxidation Increases Hydrogen Peroxide Generation in Mouse Liver

**FIGURE 1:**
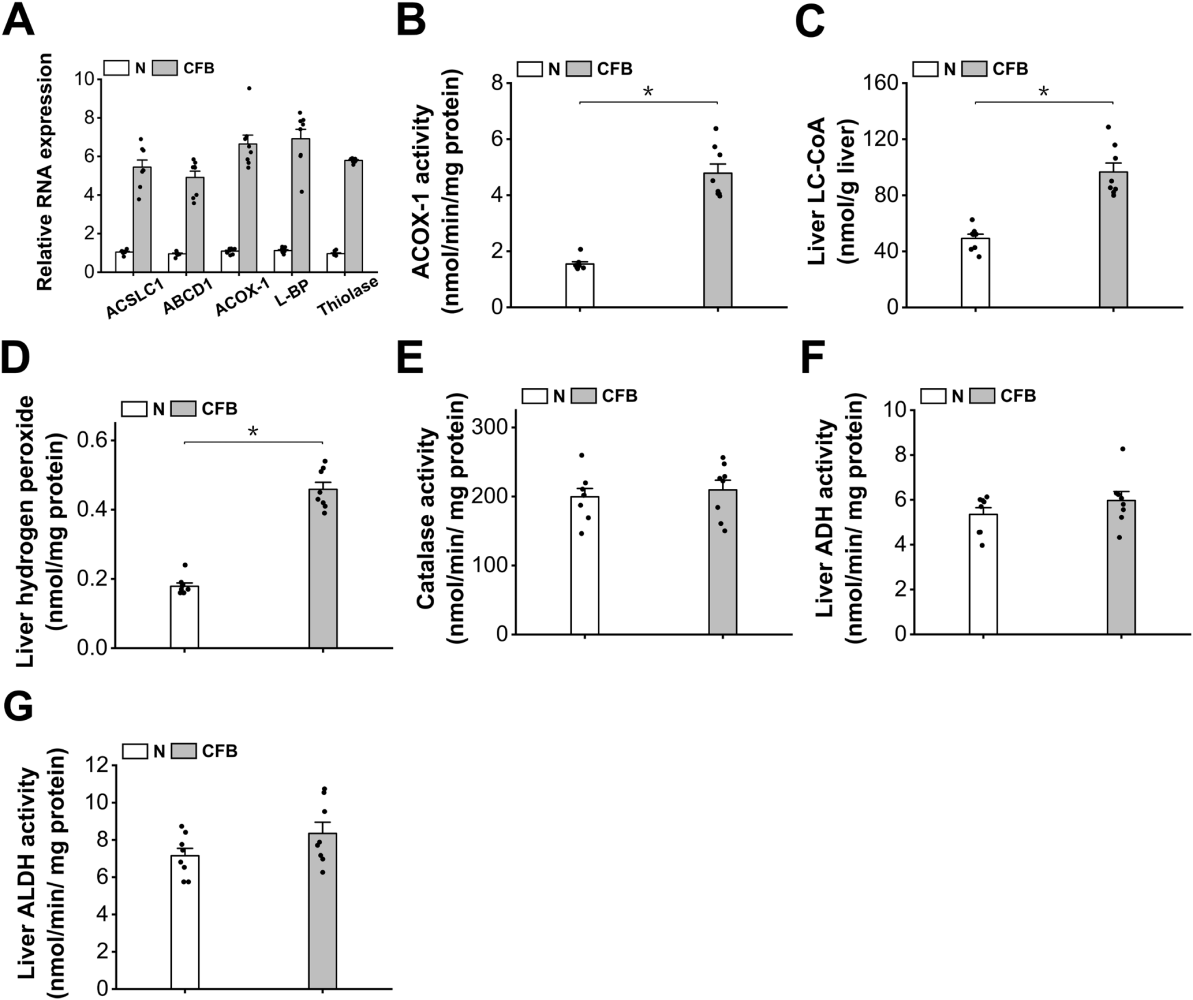
CFB treatment remarkably increases liver capacity of hydrogen peroxide generation by inducing peroxisomal β‐oxidation. (A) mRNA expressions of the enzymes involved in peroxisomal β‐oxidation were strongly induced in the liver of the mice treated with CFB. (B) CFB treatment remarkably increased liver ACOX‐1 activity in mice. (C) Liver LC‐CoA content increased significantly in the mice treated with CFB. (D) Administration of CFB considerably increased liver hydrogen peroxide formation in mice. (E) Liver catalase activity was not affected in the CFB‐treated mice. (F) Administration of CFB showed no significant effect on liver ADH activity in the mice. (G) Administration of CFB showed no significant effect on liver ALDH activity in the mice. *p < 0.05 by *t*‐test between paired groups. *n* = 8.

As a well‐known peroxisome proliferator activator receptor α isoform (PPARα) agonist, clofibrate (CFB) is applied to induce peroxisomal fatty acid β‐oxidation in mouse liver [21]. Administration of CFB to the mice strongly induced mRNA expression levels of the enzymes in peroxisomal β‐oxidation (Figure 1A). The activity of acyl‐CoA oxidase‐1 (ACOX‐1), the rate‐limiting enzyme in peroxisomal β‐oxidation, increased significantly in the liver of the CFB‐treated mice, as shown in Figure 1B. Liver long‐chain acyl‐CoA (LC‐CoA) increased abundantly in the mice treated with CFB (Figure 1C), which provided adequate substrates for peroxisomal β‐oxidation. Liver hydrogen peroxide levels were measured, and the results indicated that the treatment of CFB significantly increased liver hydrogen peroxide formation (Figure 1D). The accumulation of hydrogen peroxide after treatment with CFB was not attributed to alteration in catalase, as the activity of catalase was not affected by CFB, as shown in Figure 1E. Liver ADH and aldehyde dehydrogenase (ALDH) were not affected in the mice treated with CFB (Figure 1F,G).

### 3.2 Upregulation of Peroxisomal β‐Oxidation Accelerated Liver Metabolism of Ethanol

**FIGURE 2:**
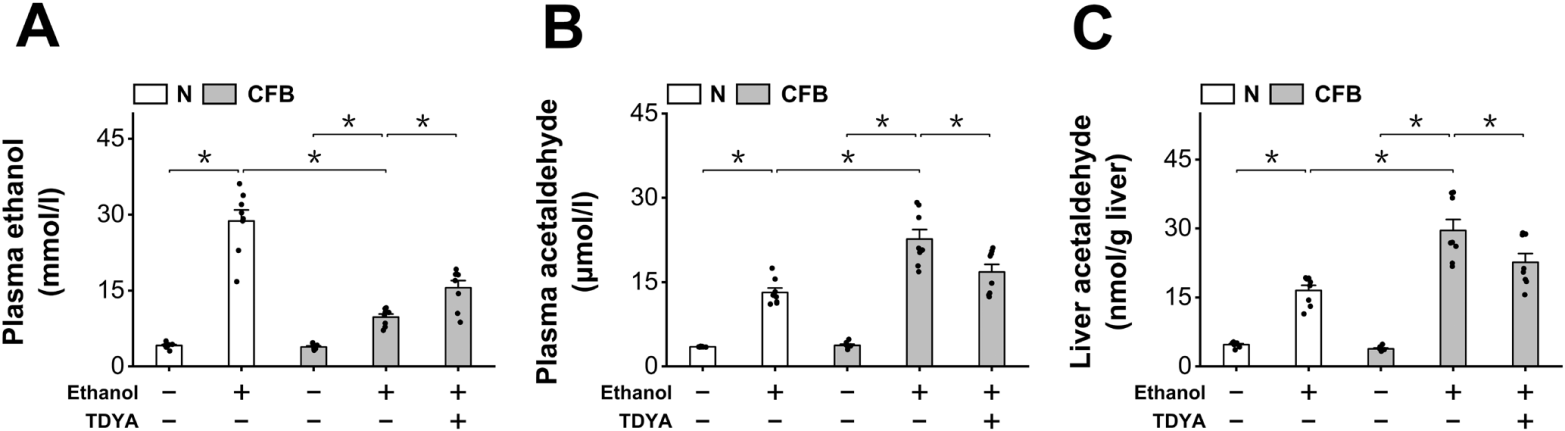
Induction of peroxisomal β‐oxidation by CFB accelerated liver metabolism of ethanol. (A) plasma level of ethanol was significantly higher in the mice after ethanol ingestion, as lowered by CFB treatment and recovered by pretreatment with TDYA. (B) plasma acetaldehyde level was significantly higher in the mice after ethanol ingestion, which was further increased by CFB treatment and reduced by pretreatment with TDYA. (C) CFB treatment caused a further increase in liver acetaldehyde in the mice treated with ethanol. which was lowered by pretreatment with TDYA. *p < 0.05 by *t*‐test between paired groups. *n* = 8.

**FIGURE 3:**
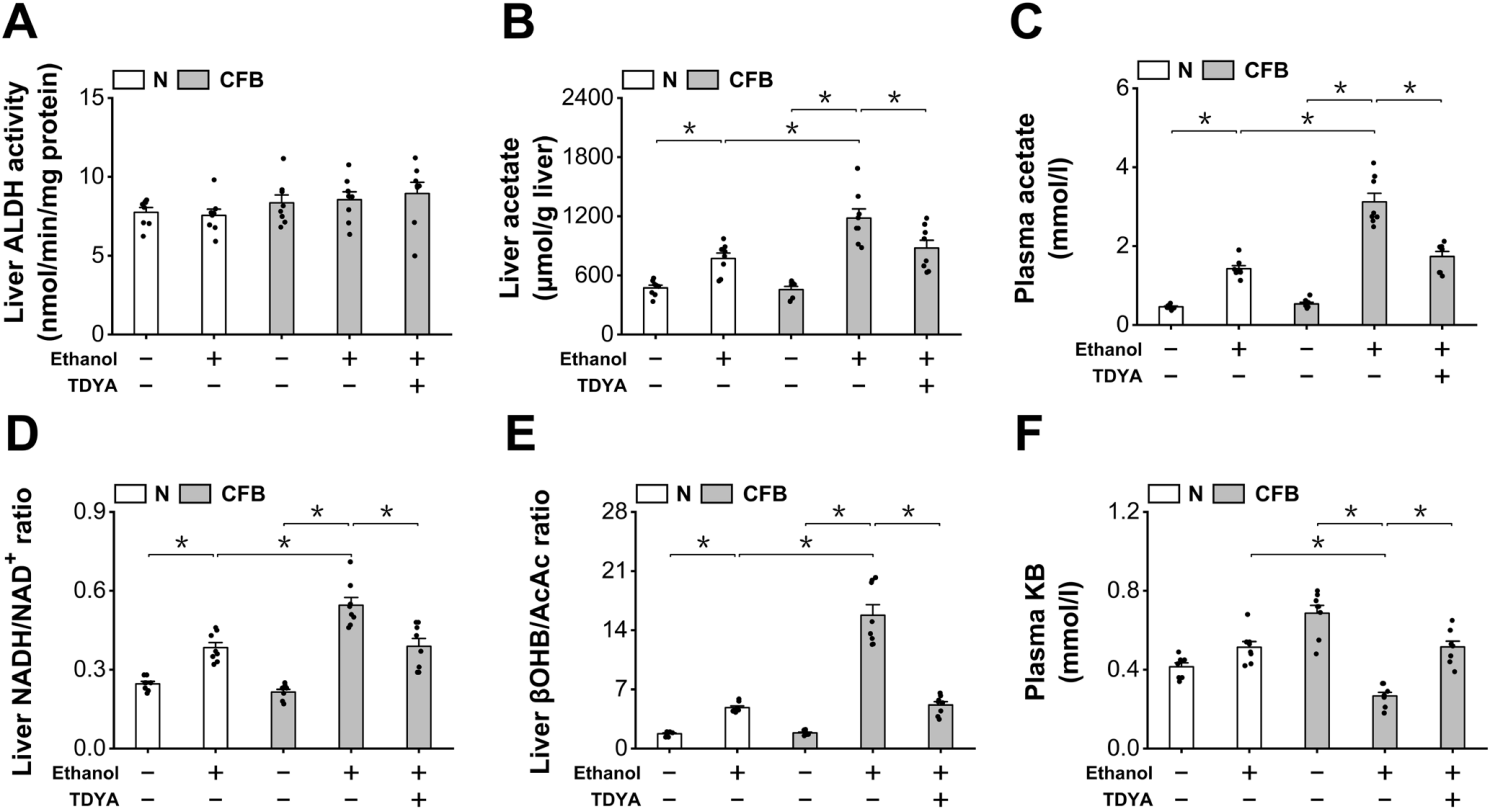
Increased oxidation of acetaldehyde causes elevation in mitochondrial NADH/NAD^+^ ratio. (A) liver ALDH activity was not changed significantly among all the groups. (B) Ethanol ingestion significantly increased liver acetate content, as further increased in the mice administered with CFB and recovered by pretreatment with TDYA. (C) Plasma acetate increased significantly in the ethanol treated mice, as further enhanced in the mice treated with CFB and lowered by TDYA. (D) Administration of ethanol significantly increased liver NADH/NAD^+^ ratio in the mice, as further enhanced in the CFB‐treated mice and reduced in the mice pretreated with TDYA. (E) Ingestion of ethanol significantly increased liver βOHB/AcAc ratio in the mice, as robustly elevated in the mice treated with CFB and lowered by pretreatment with TDYA. (F) CFB treatment significantly lowered plasma level of ketone body in the mice after ethanol ingestion, which was recovered by pretreatment with TDYA. *p < 0.05 by *t*‐test between paired groups. *n* = 8.

To investigate the impact of peroxisomal β‐oxidation on ethanol metabolism, ethanol was administered to the mice treated with CFB. 10,12‐tricosadiynoic acid (TDYA), a specific inhibitor of peroxisomal β‐oxidation [23], was administered to the CFB‐treated mice after ethanol ingestion to determine whether suppression of peroxisomal β‐oxidation might affect the liver metabolism of ethanol. Ethanol ingestion significantly increased plasma ethanol level in the mice, which was suppressed in the mice treated with CFB. Pretreatment with TDYA caused an increase in plasma ethanol level in the CFB‐treated mice after ingestion of ethanol (Figure 2A). In the meantime, plasma and liver levels of acetaldehyde were significantly higher in the mice after ethanol ingestion, which were further increased by CFB treatment and reduced by pretreatment with TDYA (Figure 2B,C). Liver ALDH activity was not significantly changed among all the groups (Figure 3A). Administration of ethanol significantly increased the generation of acetate, as reflected by the increase in liver and plasma content of acetate, which were further increased in the CFB‐treated mice and decreased by TDYA (Figure 3B,C). Liver NADH/NAD^+^ ratio was significantly higher in the mice receiving ethanol, as further elevated by the treatment with CFB and reduced by TDYA (Figure 3D). Ethanol ingestion caused a slight increase in liver β‐hydroxybutyrate (βOHB)/acetoacetate (AcAc) ratio compared with the normal group, which was robustly elevated in the CFB‐treated mice and recovered by pretreatment with TDYA (Figure 3E). As a measure of mitochondrial fatty acid β‐oxidation, plasma ketone body (KB) was then measured, and CFB treatment caused a significant decrease in plasma ketone body in the ethanol treated mice, which was increased by TDYA (Figure 3F). Therefore, the results suggested that upregulation of peroxisomal β‐oxidation accelerated ethanol metabolism and caused suppression of mitochondrial FAO through elevating mitochondrial NADH/NAD^+^ ratio.

### 3.3 Accelerated Metabolism of Ethanol by CFB Resulted in TG Accumulation

**FIGURE 4:**
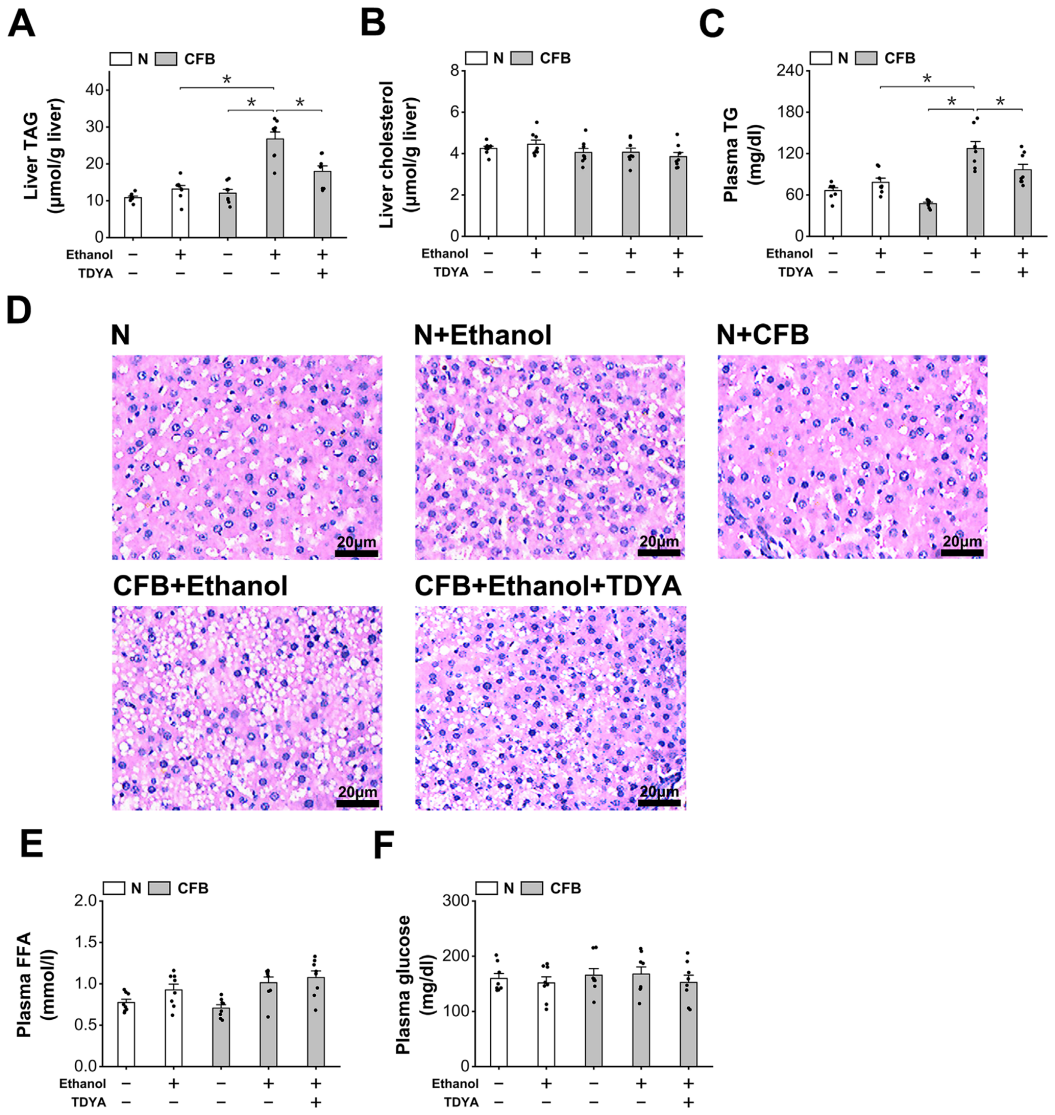
Accelerated metabolism of ethanol causes TG accumulation in the CFB‐treated mice. (A) CFB treatment remarkably increased liver TG content in the mice treated with ethanol, which was decreased by TDYA. (B) liver cholesterol was not altered among all the groups. (C) CFB treatment caused a significantly elevation in plasma TG in the mice treated with ethanol, as lowered by pretreatment with TDYA. (D) CFB treatment significantly increased liver TG content in the ethanol treated mice, which was reduced by TDYA. Magnification: X200. (E) plasma FFA level was not significantly altered among all the groups. (F) plasma glucose was not changed significantly among all the groups. *p < 0.05 by *t*‐test between paired groups. *n* = 8.

Treatment of CFB led to liver accumulation of TG in the ethanol‐treated mice, which was reduced by TDYA, as shown in Figure 4A. Liver cholesterol content was not significantly altered among all the groups (Figure 4B). Administration of CFB also significantly increased plasma TG content in the ethanol‐treated mice, as decreased by pretreatment with TDYA (Figure 4C). Liver histological analysis indicated that administration of CFB remarkably increased lipid droplets in the mice treated with ethanol, which was reduced by pretreatment with TDYA, as shown in Figure 4D. Plasma FFA and glucose were not significantly altered among all the groups, as shown in Figure 4E,F. The results indicated that alcohol ingestion caused hepatic TG accumulation in the mice treated with CFB through elevating mitochondrial NADH/NAD^+^ ratio and suppression of mitochondrial FAO. Induction of peroxisomal β‐oxidation plays a role in ethanol‐induced hepatic TG deposition.

### 3.4 Induction of Peroxisomal β‐Oxidation Increased Hydrogen Peroxide Generation in the Liver of the Fasting Mice

**FIGURE 5:**
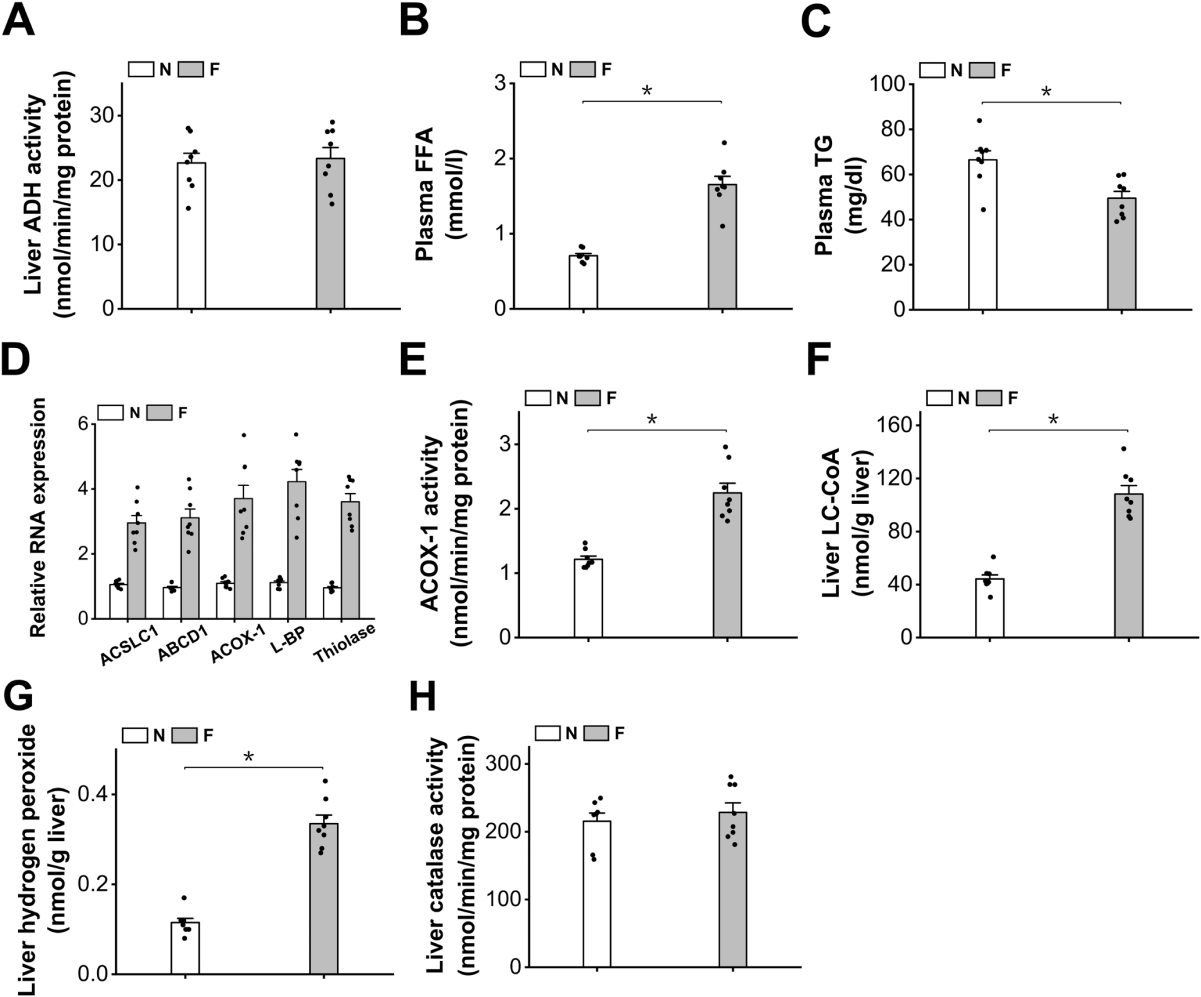
Prolonged fasting increases liver hydrogen peroxide formation through upregulation of peroxisomal β‐oxidation. (A) liver ADH activity was not affected by fasting. (B) plasma FFA level was elevated remarkably in the fasting mice. (C) plasma TG decreased remarkably in the fasting mice. (D) mRNA expressions of the enzymes involved in peroxisomal β‐oxidation were induced in the liver of the fasting mice. (E) The activity of ACOX‐1 increased significantly in the liver of the fasting mice. (F) liver LC‐CoA increased significantly in the fasting mice. (G) liver hydrogen peroxide increased significantly in the fasting mice. (H) liver catalase activity was not changed significantly in the fasting mice. *p < 0.05 by *t*‐test between paired groups. *n* = 8.

As a critical enzyme in alcohol oxidation, liver ADH activity was measured, and the results indicated that the activity of ADH was not changed in the fasting mice (Figure 5A). Plasma‐free fatty acid (FFA) increased remarkably in the mice after prolonged fasting (Figure 5B). Fasting significantly reduced plasma TG level in mice (Figure 5C). The expressions of the enzymes involved in peroxisomal β‐oxidation were upregulated in the liver of the fasting mice (Figure 5D). As expected, the activity of ACOX‐1 increased significantly in the liver of the fasting mice, as shown in Figure 5E. Fasting also caused a remarkable increase in liver LC‐CoA in the mice (Figure 5F), which provided sufficient substrates for peroxisomal β‐oxidation. Liver hydrogen peroxide was significantly higher in the fasting mice compared with the normal mice (Figure 5G). Liver catalase was not affected by fasting, as shown in Figure 5G. The results indicated that prolonged fasting stimulated hydrogen peroxide formation in the liver through upregulation of peroxisomal fatty acid oxidation.

### 3.5 Induction of Peroxisomal β‐Oxidation Stimulated Ethanol Metabolism in the Fasting Mice

**FIGURE 6:**
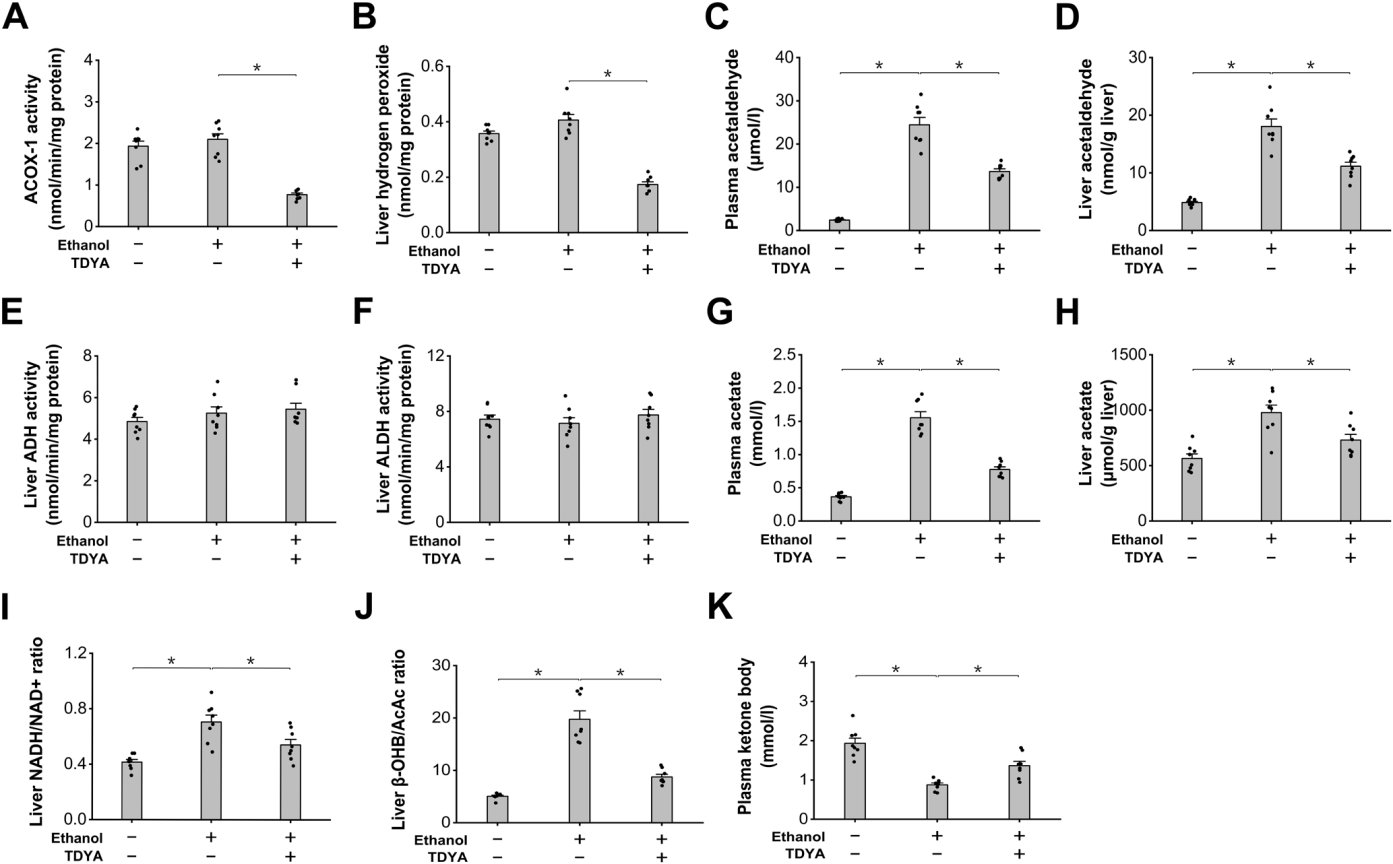
Induction of peroxisomal β‐oxidation stimulated ethanol metabolism in the fasting mice (A) TDYA treatment remarkably suppressed the activity of ACOX‐1 in the fasting mice. (B) Administration of TDYA significantly reduced hydrogen peroxide generation in liver of the fasting mice. (C) Ethanol ingestion significantly increased plasma acetaldehyde level in the fasting mice, which was reduced by pretreatment of TDYA. (D) Liver acetaldehyde level was significantly higher in the fasting mice treated with ethanol, as lowered by pretreatment of TDYA. (E) Liver ADH activity was not changed significantly in the fasting mice treated with ethanol or TDYA. (F) Liver ALDH activity was not altered significantly among all the groups. (G) Ingestion of ethanol significantly increased plasma acetate level in the fasting mice, as reduced by the pretreatment of TDYA. (H) Ethanol ingestion significantly increased liver acetate level, as lowered by pretreatment of TDYA. (I) Liver NADH/NAD^+^ ratio increased significantly in the fasting mice treated with ethanol, which was recovered by TDYA. (J) Ingestion of ethanol remarkably increased liver βOHB/AcAc ratio in the fasting mice, which was lowered by pretreatment of TDYA. (K) Ingestion of ethanol significantly lowered plasma ketone body in the fasting mice, as recovered by TDYA. *p < 0.05 by *t*‐test between paired groups. *n* = 8.

Liver ACOX‐1 activity was measured, and the results indicated that administration of TDYA significantly suppressed the activity of ACOX‐1 in the liver of the fasting mice (Figure 6A). As expected, administration of TDYA resulted in reduced hydrogen peroxide formation in the liver of the fasting mice, as shown in Figure 6B. Ethanol was then administered to the fasting mice to investigate the effect of TDYA on ethanol metabolism and hepatic lipid homeostasis. Ingestion of ethanol significantly elevated plasma and liver levels of acetaldehyde in the fasting mice, which were reduced by pretreatment with TDYA (Figure 6C,D). The activities of liver ADH and ALDH were not statistically different among all the groups (Figure 6E,F). Ethanol ingestion significantly increased plasma and liver contents of acetate compared with the fasting control, which were reduced by the treatment with TDYA, as shown in Figure 6G,H. Administration of ethanol to the fasting mice led to a significant increase in the liver NADH/NAD^+^ ratio, which was decreased by pretreatment with TDYA (Figure 6I). The liver ratio of βOHB/AcAc was measured, which was elevated remarkably in the fasting mice treated with ethanol and lowered by TDYA (Figure 6J). Plasma ketone body was also determined; the results indicated that ethanol ingestion significantly decreased plasma ketone body in the fasting mice, as increased by pretreatment with TDYA, as shown in Figure 6K.

### 3.6 Peroxisomal β‐Oxidation Plays a Role in Ethanol Induced Hepatic Lipid Accumulation in the Fasting Mice

**FIGURE 7:**
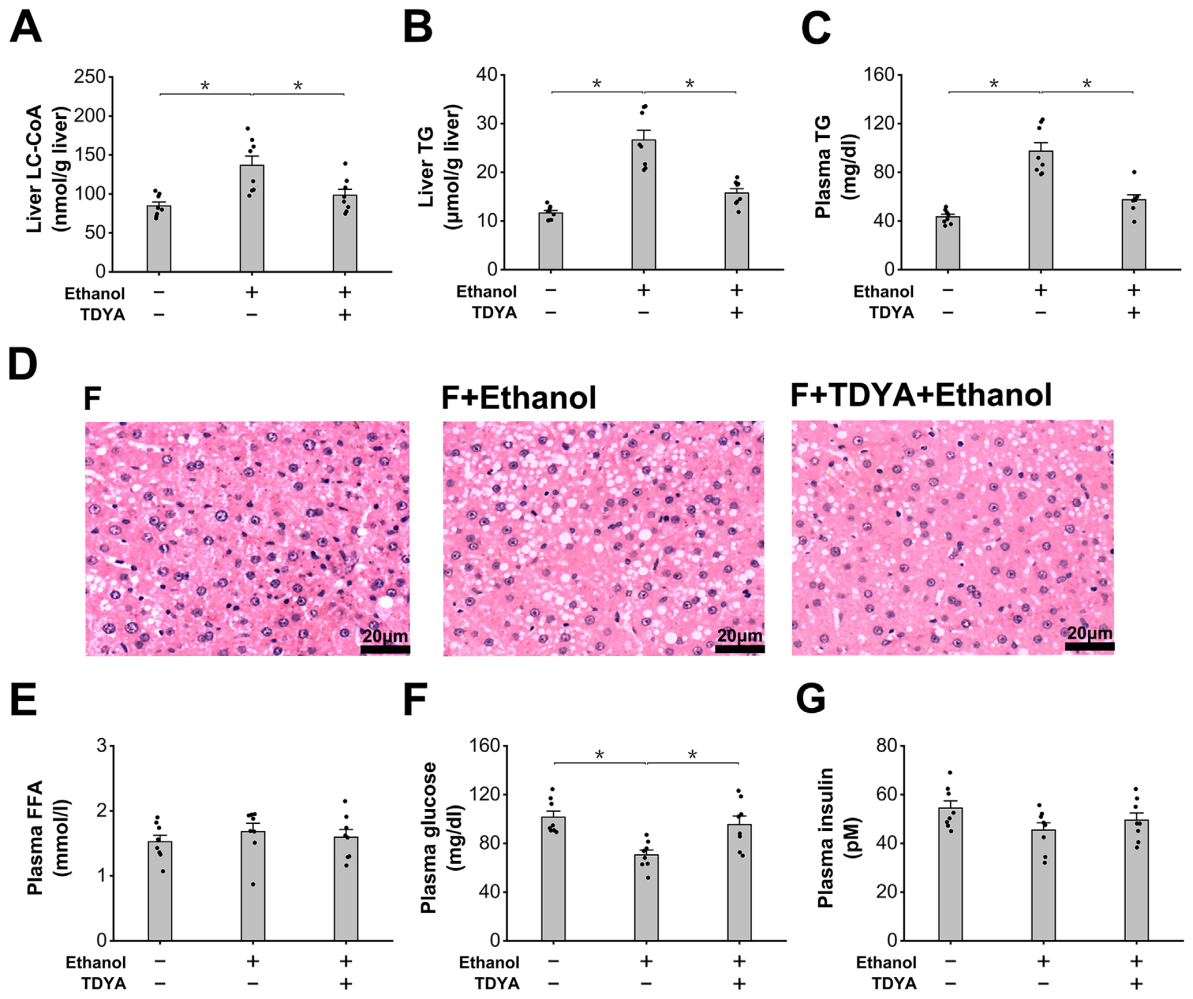
Inhibition of peroxisomal β‐oxidation improves ethanol induced hepatic steatosis in the fasting mice (A) liver LC‐CoA increased significantly in the fasting mice treated with ethanol, which was reduced by pretreatment of TDYA. (B) Ethanol ingestion remarkably increased liver TG content in the fasting mice, as reduced by pretreatment of TDYA. (C) Administration of ethanol significantly elevated plasma TG level in the fasting mice, as lowered by pretreatment of TDYA. (D) Pretreatment of TDYA improved ethanol induced hepatic steatosis in the fasting mice. Magnification: X200. (E) Plasma FFA level was not altered significantly among all the groups. (F) Ingestion of ethanol decreased plasma glucose in the fasting mice, which was recovered by pretreatment of TDYA. (G) Plasma insulin level was not changed significantly among all the groups. *p < 0.05 by *t*‐test between paired groups. *n* = 8.

Ethanol ingestion resulted in a significant increase in liver LC‐CoA in the fasting mice, which was reduced by TDYA (Figure 7A). Administration of ethanol also caused a significant elevation in liver and plasma levels of TG compared with the fasting control, which were lowered by pretreatment with TDYA, as shown in Figure 7B,C. Liver sections indicated that ethanol ingestion led to a significant increase in lipid droplets in the liver of the fasting mice, while suppression of peroxisomal β‐oxidation by TDYA improved ethanol‐induced hepatic steatosis in the fasting mice (Figure 7D). Plasma FFA was not significantly changed among all the groups (Figure 7E). Ethanol treatment significantly decreased plasma glucose in the fasting mice, as recovered by pretreatment with TDYA (Figure 7F). Plasma insulin levels were not significantly altered in fasting mice after treatment with ethanol or TDYA, as shown in Figure 7G. The results suggested that induction of peroxisomal β‐oxidation plays a critical role in ethanol‐induced hepatic TG accumulation in animals under a fasted state, while specific inhibition of ACOX‐1, the first enzyme in peroxisomal β‐oxidation that generates hydrogen peroxide, improved alcohol‐induced hepatic steatosis in the fasting mice.

## 4 Discussion

**FIGURE 8:**
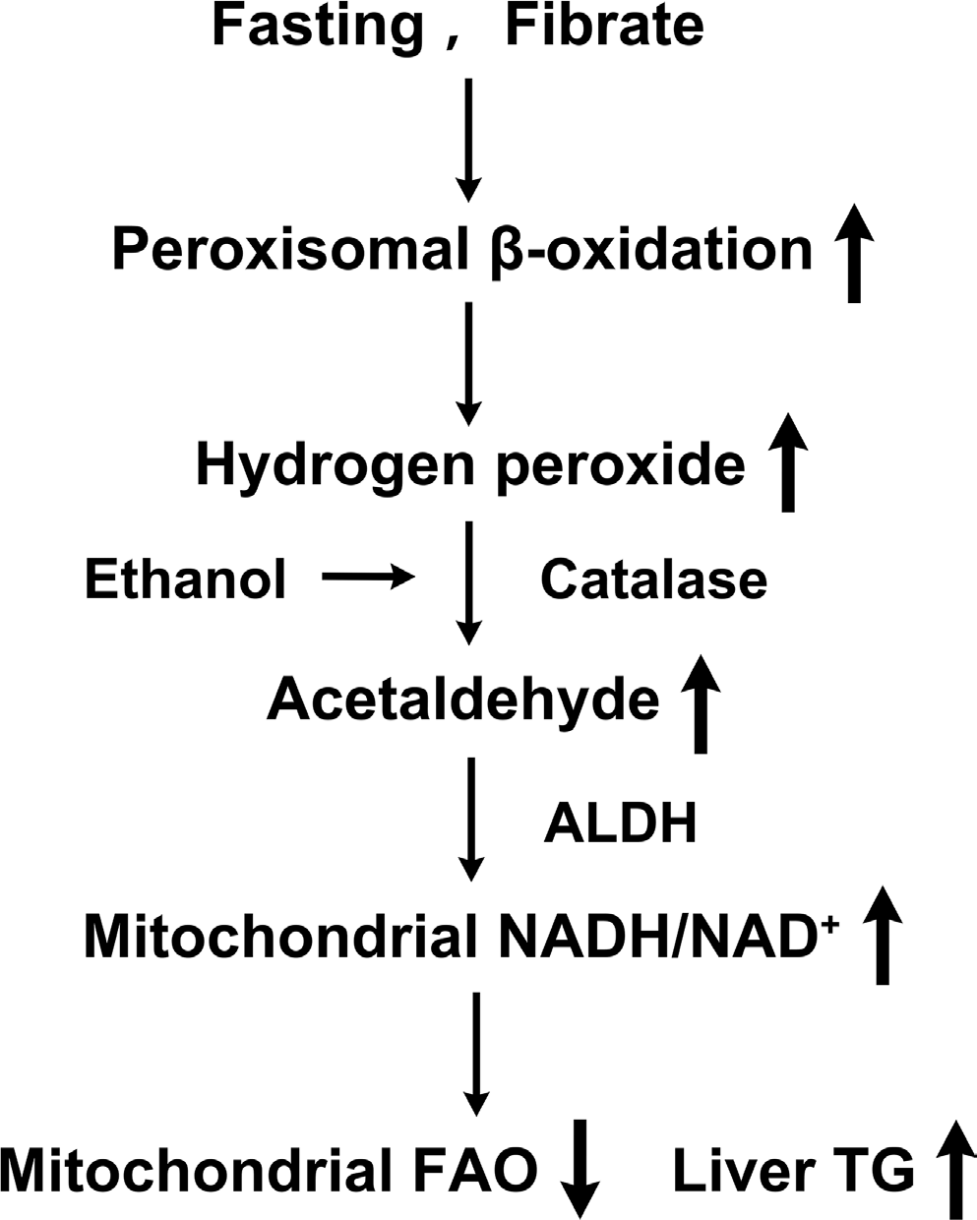
Proposed mechanism by which induction of peroxisomal β‐oxidation stimulates ethanol metabolism and causes fatty liver in the fasting and fibrate‐treated animals.

Alcohol ingestion is well known to be able to induce fatty liver and related liver injury [1, 2]; however, the potential metabolic pathways and precise mechanism by which metabolism of ethanol causes suppression of mitochondrial FAO and TG accumulation are not fully illustrated. The study reveals a novel role of peroxisomal fatty acid oxidation in the metabolism of ethanol as well as ethanol‐induced hepatic lipid accumulation in animals under a ketogenic state. The proposed mechanism is shown in Figure 8. Prolonged fasting and fibrate treatment cause increased hepatic uptake of fatty acids and induction of peroxisomal β‐oxidation. Upregulation of peroxisomal β‐oxidation results in excessive generation of hydrogen peroxide, which stimulates ethanol metabolism and significantly increases the formation of acetaldehyde; further oxidation of acetaldehyde into acetate remarkably elevates the mitochondrial ratio of NADH/NAD^+^ and causes suppression of mitochondrial FAO, which ultimately leads to the accumulation of TG in the liver. This study gives us a better understanding of the oxidative mechanism of ethanol and the pathogenesis of alcoholic fatty liver and related diseases.

Ethanol is primarily metabolized in the liver, which is firstly metabolized to acetaldehyde and further oxidized to acetate by mitochondrial ALDH [34]. There are three major enzymatic systems that oxidize ethanol to acetaldehyde, including ADH, catalase, and microsomal ethanol oxidation system (MEOS). It is generally considered that the ADH pathway plays a primary role in the oxidation of ethanol to acetaldehyde; this may be true under normal physiological conditions. However, under ketogenic conditions such as prolonged fasting, the metabolism of ethanol by ADH might not play a prominent role [12, 13]. The peroxisomal catalase pathway plays a primary role in the metabolism of ethanol in fasting animals, provided the supply of free fatty acids is enough [13, 18, 19]. The results in this study suggested that the catalase pathway plays an important role in the metabolism of ethanol, and the induction of peroxisomal fatty acid oxidation by CFB provides sufficient hydrogen peroxide for the catalytic reaction of the catalase pathway, which accelerated ethanol clearance and led to increased hepatic TG levels. Specific suppression of peroxisomal acyl‐CoA oxidase by TDYA significantly reduced the generation of hydrogen peroxide, which suppressed ethanol oxidation and reduced hepatic TG levels in the fasting mice. Our results well support the proposed mechanism that the metabolism of ethanol by the catalase pathway will be significant when the supply of hydrogen peroxide is sufficient. MEOS is mainly derived from the smooth endoplasmic reticulum (ER) in the liver, which is capable of converting ethanol into acetaldehyde in the presence of NADPH and oxygen [35]. The metabolism of ethanol by MEOS is controversial; evidence indicated that the oxidation of ethanol by the NADPH‐dependent MEOS is due to hydrogen peroxide formation from NADPH and a subsequent peroxidation of ethanol by contaminating catalase [12, 36, 37]. Furthermore, it was reported that the removal of physiological concentrations of alcohol by the liver in normal rats was almost completely inhibited by pyrazole, an inhibitor for ADH that showed no inhibitory activity toward MEOS [38]. Therefore, it is likely that MEOS might not play a prominent role in ethanol metabolism under physiological conditions.

Besides being metabolized in the liver, a recent study suggests that the intestine might play an important role in promoting acetaldehyde metabolism and clearance. Liver‐derived acetaldehyde is excreted via bile into the gastrointestinal tract, which is further metabolized into acetate by intestinal epithelial ALDH2, thereby reducing the systemic level of acetaldehyde. Modulation of bile flow dynamically regulates serum acetaldehyde concentrations and subsequent alcohol‐drinking behaviors. Therefore, it is suggested that the detoxification of alcohol is coordinately driven by the gut–liver axis [39].

Excessive oxidation of ethanol has been well known to induce hepatic lipid deposition, while the potential pathogenic mechanism is not fully demonstrated. Possible mechanisms involve suppression of mitochondrial FAO, stimulation of fatty acid synthesis, inhibition of AMPK, activation of SREBP‐1c, and inhibition of PPARα [6, 40]. The most probable mechanism for the acute effect of ethanol on hepatic TG accumulation is suppression of mitochondrial FAO through elevation in the intramitochondrial NADH/NAD^+^ ratio [41, 42], which results in an increased availability of long‐chain free fatty acids, thereby enhancing esterification and leading to accumulation of liver TG [6, 7]. The elevation in the mitochondrial NADH/NAD^+^ ratio as caused by ethanol metabolism might be ascribed to cytosolic oxidation of ethanol by ADH or mitochondrial oxidation of acetaldehyde by ALDH. In the cytosol, ADH metabolizes ethanol to acetaldehyde and reduces NAD^+^ to NADH, which significantly increases the cytosolic NADH/NAD^+^ ratio and might further affect mitochondrial NADH through redox shuttles. However, the effect may not be significant under the condition of prolonged fasting when the mitochondrial redox state of NADH is already high, thus the cytosolic NADH/NAD^+^ is not a controlling factor affecting lipid accumulation in the fasted livers. We suggest that the oxidation of acetaldehyde to acetate might play a key role in suppressing mitochondrial FAO through directly elevating the mitochondrial NADH/NAD^+^ ratio. Previous reports suggested that high concentrations of acetaldehyde showed significant inhibitory effects on fatty acid β‐oxidation in isolated liver mitochondria, possibly through suppression of the activity of the TCA cycle and respiration chain [43, 44]. Our results suggest that induction of peroxisomal fatty acid oxidation by CFB treatment or fasting significantly accelerates ethanol oxidation by the peroxisomal catalase system through increasing the supply of hydrogen peroxide; the generated acetaldehyde subsequently enters into the mitochondria and elevates the mitochondrial NADH/NAD^+^ ratio through oxidation by ALDH, which ultimately results in suppression of mitochondrial FAO and causes TG accumulation.

In contrast to the effects of peroxisomal β‐oxidation in binge ethanol‐induced hepatic lipid accumulation, induction of peroxisomal β‐oxidation is protective against the alcoholic fatty liver in chronic models [45]. It is suggested that a critical point that leads to the discrepancy is the dose of the ethanol used in the experiments. In the chronic model, the dose of ethanol is moderate, which results in mild suppression of mitochondrial FAO [46], and peroxisomal β‐oxidation is activated as an alternative pathway, partially compensating for the impairment of mitochondrial function and reducing hepatic lipid accumulation. While in this study, acute administration of a large dose of ethanol in the mice pretreated with PPARα agonist considerably elevates mitochondrial NADH/NAD^+^ ratio and causes serious suppression of mitochondrial β‐oxidation and hepatic lipid deposition.

The peroxisomal fatty acid β‐oxidation system was discovered in mammalian peroxisomes in the mid‐1970s [47], which was responsible for the metabolism of long‐chain and very long‐chain fatty acids. ACOX‐1 is a flavoenzyme that catalyzes the initial and rate‐determining reaction of peroxisomal β‐oxidation using straight‐chain fatty acyl‐CoAs as the substrates, which donates electrons to molecular oxygen, generating hydrogen peroxide. Therefore, peroxisomal ACOX‐1 plays a role in ethanol oxidation by providing hydrogen peroxide to the catalase–hydrogen peroxide complex system. The catalase pathway of ethanol metabolism will be more prominent under the conditions of fasting, diabetes, high‐fat diet feeding, and hypolipidemic drug treatment when peroxisomal β‐oxidation is upregulated [17], which will accelerate ethanol oxidation and acetaldehyde release. In this study, administration of CFB remarkably induced peroxisomal ACOX‐1 activity and increased hydrogen peroxide generation, which strongly stimulated ethanol oxidation. Conversely, specific inhibition of ACOX‐1 by TDYA reduced hydrogen peroxide formation and suppressed ethanol oxidation in fasting mice. It is notable that 4‐methylpyrazole, a known inhibitor of ADH that abolished ethanol‐induced hepatic TG accumulation in animals, also showed inhibitory activity against peroxisomal FAO [48].

For a long time, the pathophysiological roles of the peroxisomal FAO system are not clear until recent years; evidences suggest that this fatty acid metabolism system might play a role in regulating mitochondrial FAO and inducing hepatic steatosis through the metabolites acetyl‐CoA and hydrogen peroxide [26, 49, 50, 51, 52]. However, whether this metabolism system might play a regulatory role in ethanol‐induced hepatic TG accumulation under physiological conditions is not addressed. This study demonstrates a role of peroxisomal FAO in the metabolism of ethanol in animals, and the results suggest that upregulation of ACOX‐1, the first and rate‐limiting enzyme in peroxisomal β‐oxidation, stimulates ethanol oxidation by increasing the supply of hydrogen peroxide for the catalase reaction, which accelerates acetaldehyde formation and leads to hepatic TG accumulation through elevating mitochondrial NADH/NAD^+^ ratio and suppression of mitochondrial FAO. We suggest that the induction of peroxisomal β‐oxidation acts as a critical mechanism for alcohol‐induced hepatic lipid accumulation under ketogenic conditions.

Diabetes and obesity have been well known to induce liver peroxisomal fatty acid β‐oxidation in the livers of animals as well as human beings [17, 53]; especially, solid evidence indicates that peroxisomal fatty acid β‐oxidation is upregulated in the liver of patients with alcoholic liver disease [54]. The enhanced metabolic capacity of ethanol through the catalase pathway will accelerate the generation of the toxic molecule acetaldehyde and the development of alcohol fatty liver and related liver injury in diabetic and obese individuals. Besides, fibrate drugs are widely used for the treatment of hyperlipidemia; as peroxisomal ACOX‐1 is strongly induced by this kind of drug, we proposed that diabetic patients with severe obesity administered fibrate drugs might have a high risk of alcohol‐associated liver diseases when ingesting excessive alcohol, as hyperinduction of peroxisomal β‐oxidation will greatly enhance the turnover rate of ethanol and accelerate the onset and development of alcoholic fatty liver and related liver injury. As induction of peroxisomal fatty acid β‐oxidation emerges as a pathogenic mechanism for ethanol‐induced hepatic lipid accumulation, the key enzyme ACOX‐1 in peroxisomal fatty acid oxidation might be a potential drug target for the treatment of alcoholic fatty liver by lowering the level of ethanol oxidation. In this study, specific suppression of ACOX‐1 by a specific inhibitor TDYA significantly reduced liver generation of hydrogen peroxide, which suppressed ethanol metabolism and improved hepatic steatosis in fasting mice. The results provide evidence that small molecules specifically targeting ACOX‐1 might be a potential pathway in treating alcohol‐induced fatty liver by suppressing peroxisomal oxidation of ethanol.

## Author Contributions

**Yida Zhang, Wei Zhang:** investigation, methodology, formal analysis. **Yicong Li, Haoya Yao:** investigation, methodology. **Yaoqing Wang, Xiao Zhang:** investigation. **Jia Zeng:** conceptualization, methodology, supervision, writing – reviewing and editing, funding acquisition, project administration.

## Conflicts of Interest

The authors declare no conflicts of interest.

## Data Availability

All data are contained within the manuscript.
